# Number of Directions Determined by a Set in $\mathbb {F}_{q}^{2}$ and Growth in $\mathrm {Aff}(\mathbb {F}_{q})$

**DOI:** 10.1007/s00454-021-00284-6

**Published:** 2021-03-08

**Authors:** Daniele Dona

**Affiliations:** 1 Mathematisches Institut, Georg-August-Universität Göttingen, Bunsenstraße 3-5, 37073 Göttingen, Germany; 2 Present Address: Einstein Institute of Mathematics, The Hebrew University of Jerusalem, Edmond J. Safra Campus Givat Ram, Jerusalem, 9190401 Israel

**Keywords:** Affine group, Directions, Finite plane, Growth, 52C10, 52C30, 20F69, 20E34, 12E10

## Abstract

We prove that a set *A* of at most *q* non-collinear points in the finite plane $\mathbb {F}_{q}^{2}$ spans more than ${|A|}/\!{\sqrt{q}}$ directions: this is based on a lower bound by Fancsali et al. which we prove again together with a different upper bound than the one given therein. Then, following the procedure used by Rudnev and Shkredov, we prove a new structural theorem about slowly growing sets in $\mathrm {Aff}(\mathbb {F}_{q})$ for any finite field $\mathbb {F}_{q}$, generalizing the analogous results by Helfgott, Murphy, and Rudnev and Shkredov over prime fields.

## Introduction

Among the many different problems related to the study of growth and expansion in finite groups, the study of the affine group over finite fields has occupied a particularly interesting place. The affine group

$$\begin{aligned} \mathrm {Aff}(\mathbb {F})=\left\{ \begin{pmatrix}a&{}b\\ 0&{}1\end{pmatrix}\, \bigg |\;a\in \mathbb {F}^{*},b\in \mathbb {F}\right\} , \end{aligned}$$

where $\mathbb {F}$ is a finite field, is one of the smallest interesting examples of an infinite family of finite groups on which questions of growth of sets $A\subseteq \mathrm {Aff}(\mathbb {F})$ can yield nontrivial answers, and it has been used to showcase techniques applicable to more general situations, like the pivot argument; on the other hand, its shape makes it uniquely suitable to study the so-called sum-product phenomenon, related to growth of sets inside finite fields under both addition and multiplication. For both of these points of view, a remarkable example is provided in Helfgott’s survey [9, § 4.2].

Structural theorems about growth in $\mathrm {Aff}(\mathbb {F}_{p})$ (*p* prime) have been produced in the last few years, describing in substance what a set *A* with small growth must look like. Results like Helfgott’s [9, Prop. 4.8] and Murphy’s [14, Thm. 27] belong to a first generation of proofs that rely, in one way or another, on sum-product estimates; they already accomplish the goal of characterizing quite well a slowly growing *A*: such a set must essentially either be a point stabilizer or be contained in a few vertical lines, which in addition get filled in finitely many steps if |*A*| is at least of the same order of magnitude as *p*.

Rudnev and Shkredov [16] have then quantitatively improved this classification in $\mathrm {Aff}(\mathbb {F}_{p})$: the main attractivity of their result, however, resides in the fact that they avoid any explicit ties to sum-product results. What they rely on instead is a geometric theorem by Szőnyi [19, Thm. 5.2] that gives a good lower bound on the number of directions spanned by a set of non-collinear points in the plane $\mathbb {F}_{p}^{2}$ for *p* prime: this approach can pave the way to a future new generation of efforts.

Following the approach by Rudnev and Shkredov, we first produce an analogous version of Szőnyi’s result for the plane $\mathbb {F}_{q}^{2}$, where *q* is any prime power; then we use that estimate to prove a structural theorem on slowly growing sets in $\mathrm {Aff}(\mathbb {F}_{q})$ (resembling the corresponding ones for $\mathrm {Aff}(\mathbb {F}_{p})$ mentioned before), which to the best of our knowledge is the first of its kind.

Throughout the paper, *p* will always denote a prime and *q* a power of *p*. We occasionally make use of the big *O* and big $\Omega$ notations, the latter following Knuth’s convention [12]; an index $O_{\varepsilon },\Omega _{\varepsilon }$ indicates that the implicit constant may depend on the variable $\varepsilon$.

Given a set *A* inside the plane $\mathbb {F}^{2}$, the set of *directions* spanned or determined by *A* denotes the set

$$\begin{aligned} D=\left\{ \frac{b'-b}{a'-a}\;\bigg |\;(a,b),(a',b')\in A, \; (a,b)\ne (a',b')\right\} \subseteq \mathbb {F}\cup \{\infty \}, \end{aligned}$$

where conventionally $\infty$ corresponds to the fraction with $a'-a=0$. We make free use of the natural identification $\mathrm {Aff}(\mathbb {F})\leftrightarrow \mathbb {F}^{*}\times \mathbb {F}$ given by

$$\begin{aligned} \begin{pmatrix}a&{}b\\ 0&{}1\end{pmatrix}\in \mathrm {Aff}(\mathbb {F}) \ \ \ \ \ \longleftrightarrow \ \ \ \ \ (a,b)\in \mathbb {F}^{*}\times \mathbb {F} \end{aligned}$$

so that we may refer to points, lines, and directions even when speaking about the group $\mathrm {Aff}(\mathbb {F})$; in particular, we call $\pi :\mathrm {Aff}(\mathbb {F})\rightarrow \mathbb {F}^{*}$ the map corresponding to the projection on the first component, so that the preimage of a point through this map is a vertical line. $\mathrm {Aff}(\mathbb {F})$ acts also on $\mathbb {F}$ as $(a,b)\cdot x=ax+b$, and we think of this action when we refer to point stabilization: we call $\mathrm {Stab}(x)$ the set $\{(a,b)\mid (a,b)\cdot x=x\}$, and we note that this set too describes a line in $\mathbb {F}^{2}$. Finally, *U* denotes the unipotent subgroup corresponding to $\{1\}\times \mathbb {F}$, again a vertical line.

As said before, one of the starting points of the new-style result for slowly growing sets in $\mathrm {Aff}(\mathbb {F}_{p})$ is the following bound by Szőnyi.

### Theorem 1.1

Let *p* be a prime, and let $A\subseteq \mathbb {F}_{p}^{2}$ with $1<|A|\le p$. Then either *A* is contained in a line or *A* spans $\ge ({|A|+3})/{2}$ directions.

With that, Rudnev and Shkredov prove the following (see [16, Thm. 5]).

### Theorem 1.2

Let *p* be a prime and let $A\subseteq \mathrm {Aff}(\mathbb {F}_{p})\leftrightarrow \mathbb {F}_{p}^{*}\times \mathbb {F}_{p}$ with $A=A^{-1}$ and $|A^{3}|=C|A|$. Then at least one of the following is true:

- $$A\subseteq \mathrm {Stab}(x)$$ for some $$x\in \mathbb {F}_{p}$$;
- when $1<|A|\le (1+\varepsilon ) p$ for some $0<\varepsilon <1$, we have $|\pi (A)|\le 2C^{4}$;
- when $|A|>(1+\varepsilon ) p$ for some $0<\varepsilon <1$, we have $|\pi (A)|=O_{\varepsilon }(C^{3}|A|/p)$, and, in particular, for $|A|>4p$ we have $|\pi (A)|\le {2}C^{3}|A|/p$ and $A^{8}\supseteq U$.

Szőnyi’s bound is part of a long history of applications of results about *lacunary polynomials* (i.e., polynomials made of a small number of monomials with respect to their degree) over finite fields to finite geometry: the reader interested in similar applications can check [19] and its bibliography.

Many results in this area can apply, with the appropriate modifications, to $\mathbb {F}_{q}$ as well. In this case, however, bounds on the number of directions spanned by a set in the finite plane appear to be messier, and understandably so: unlike in the case of $\mathbb {F}_{p}$, the number of directions determined by *A* tends to congregate around values ${|A|}/{p^{i}}$ for powers $p^{i}|q$; this is due to the fact that there may exist sets with multiples of $p^{i}$ points on each line that are so well structured that they sit in relatively few directions compared to the amount of points they have (see [3, §5] for an example of this assertion when $|A|=q$).

The result we essentially use, on the number of directions spanned in $\mathbb {F}_{q}^{2}$ by some set with $1<|A|\le q$, is due to Fancsali et al. [7, Thm. 17]: for the lower bound they found we give here a proof that is very similar to theirs, but we also prove a different upper bound that can be more or less advantageous than theirs depending on the situation (Theorem 2.2). Used directly, the lower bound can only give us about |*A*|/(*q*/*p*) directions; a tighter theorem, in the style of [3, Thm. 1.1], would give not only $p^{i}| p^{e}=q$, but also *i*|*e* (and therefore a much better lower bound of ${|A|}/\!{\sqrt{q}}$ directions): [3, Thm. 1.1] however works only for $|A|=q$, and the lack of a complete set of *q* points is crucial in worsening the condition on the denominator $p^{i}$ during the proof.

Nevertheless, it turns out that a simple observation *can* make us achieve the bound with $\sqrt{q}$ in the denominator: at its core, we use the fact that a set of points *A* either sits on $\ge \sqrt{q}$ parallel lines or has a line with $\ge {|A|}/\!{\sqrt{q}}$ points on it. Our first main result then, playing the role of Szőnyi’s bound in [16], is as follows.

### Theorem 1.3

Let $q=p^{e}$ be a prime power, and let $A\subseteq \mathbb {F}_{q}^{2}$ with $1<|A|\le q$. Then either *A* is contained in a line or *A* spans

- $>{|A|}/\!{\sqrt{q}}$ directions for *e* even,
- $>{|A|}/({p^{({e-1})/{2}}+1})$ directions for *e* odd.

Observe that the theorem is only a constant away from Szőnyi’s bound when we use it for $q=p$; we add that actually the proof can be easily adjusted to yield that bound exactly: we chose not to do so in order to get a cleaner statement, with case (b) valid for all odd *e*. The quantity in (b) is also larger than ${|A|}/\!{\sqrt{q}}$, in all cases except for $q\in \{2,3,8\}$: however one can again examine the proof and readily cover the three remaining values. Thus, the set *A* spans $>{|A|}/\!{\sqrt{q}}$ directions for all *q*.

Using Theorem 1.3 and following more or less the same proof as in [16], we obtain our second main result, generalizing Theorem 1.2 to any $\mathbb {F}_{q}$.

### Theorem 1.4

Let $q=p^{e}$ be a prime power and let $A\subseteq \mathrm {Aff}(\mathbb {F}_{q})\leftrightarrow \mathbb {F}_{q}^{*}\times \mathbb {F}_{q}$ with $A=A^{-1}$ and $|A^{3}|=C|A|$. Then at least one of the following is true:

- $$A\subseteq \mathrm {Stab}(x)$$ for some $$x\in \mathbb {F}_{q}$$;
- when $1<|A|\le q$ we have $|\pi (A)|<(p^{\lfloor {e}/{2}\rfloor }+2) C^{4}$, while when $q<|A|<(3+2\sqrt{2}) q$ we have $|\pi (A)|<(4+2\sqrt{2}) C^{4}$;
- when $|A|\ge (3+2\sqrt{2}) q$ we have $|\pi (A)|<{2}C^{3}|A|/q$ and $A^{8}\supseteq U$.

The statement above looks remarkably similar to Theorem 1.2, and is qualitatively as strong a structural theorem as in the case of $\mathrm {Aff}(\mathbb {F}_{p})$. The $p^{\lfloor {e}/{2}\rfloor }$ in case (b) cannot be improved in general: for *e* even, there is a natural embedding of $\mathbb {F}_{\sqrt{q}}$ inside $\mathbb {F}_{q}$, and $A=(\mathbb {F}_{\sqrt{q}})^{*}\times \mathbb {F}_{\sqrt{q}}$ has $|A|<q$, $C=1$, and $|\pi (A)|=p^{{e}/{2}}-1$. See Sect. 4 for further remarks.

Let us comment however on a small difference between Theorem 1.2 and the result for $\mathbb {F}_{p}$ featured in [16]. The case of a medium-sized *A* (i.e., $1<{|A|}/{q}=O(1)$) has been placed into alternative (c) by Rudnev and Shkredov and into alternative (b) by us, essentially losing the $A^{k}\supseteq U$ implication: this has been done because the subgroup *H* of Kneser’s theorem [11] can stifle the growth of *A*, in a way that the Cauchy–Davenport inequality ([5, Thm. VII], [6], see [20, Thm. 5.4]) could not; asking for *p* large enough is innocuous in the latter, but not in the former: see also Sect. 3 where we use it.

We could still use Alon’s bound [1, (4.2)] on the number of lines in the projective plane as done in [16], since it holds for $\mathbb {F}_{q}$ as well: this would give for example

$$\begin{aligned} |\pi (A)|<\frac{2 (\sqrt{5}+1)}{(7-3\sqrt{5}) q} C^{3}|A|\qquad \text {for}\quad |A|\ge \frac{\sqrt{5}+1}{2} q \end{aligned}$$

(where the maximum of ${\varepsilon ^{2}(1-\varepsilon )}/({2 (1+\varepsilon )})$ is located) and in general $|\pi (A)|=O_{\varepsilon }(C^{3}|A|/q)$ for $|A|\ge (1+\varepsilon ) q$; then, upon using Kneser’s theorem, one could either ask for *p* large enough ($p>100$ in the first case, say, and $p=\Omega _{\varepsilon }(1)$ in general) or classify separately the sets *A* with large *H* (which should be possible, because having large $H=\mathrm {Stab}(A^{2})$ is a rather restrictive condition to satisfy), and an additional conclusion $A^{k}\supseteq U$ for $k=O_{\varepsilon }(1)$ would be reached. It would probably be interesting to explore more deeply these medium-sized sets; however, for the purpose of obtaining a structural result like Theorem 1.4 whose numerical details are of secondary relevance, we deemed to be simpler and just as effective to reduce that case to alternative (b), especially as the observation behind our ability to do so (Lemma 2.1) is very elementary.

As a final note, we observe that some results on which we rely in the case of $\mathbb {F}_{q}^{2}$ have been studied in the case of $\mathbb {F}_{q}^{n}$ as well. For instance, Lemma 2.1 has been generalized to $\mathbb {F}_{q}^{n}$ in [10], which also deals with directions determined by two different sets, and generalizations of Proposition 3.1 appear in [13, 21].

## Number of Directions in $\mathbb {F}_{q}^{2}$

In the present section we prove bounds about the number of directions determined by sets of points in the plane $\mathbb {F}_{q}^{2}$, which lead eventually to Theorem 1.3. Let us start with the following simple statement: it does not concern Theorem 1.3, but it will allow us in the next section to deal quickly with the sets *A* whose size is slightly larger than *q*.

### Lemma 2.1

Any set $A\subseteq \mathbb {F}_{q}^{2}$ with $|A|>q$ spans all $q+1$ directions.

### Proof

The result is immediate: by the pigeonhole principle, for any given direction, one of the *q* parallel lines in $\mathbb {F}_{q}^{2}$ following that direction has to contain at least two points of *A*.$\square$

As a complement to Lemma 2.1, the following theorem deals with the number of directions spanned by sets of size at most *q*. As remarked before, a theorem of the same nature appears already in [7], and it is proven very similarly using the same techniques deriving from the study of lacunary polynomials.

### Theorem 2.2

Let $q=p^{e}$ be a prime power, let $A\subseteq \mathbb {F}_{q}^{2}$ with $1<|A|\le q$, and let *D* be the set of directions determined by *A*. Then either $|D|=1$ (and *A* is contained in a line), $|D|=q+1$ (and *A* spans all directions), or there are two integers $0\le l_{2}\le l_{1}<e$ such that

$$\begin{aligned} |D|&\ge \frac{|A|-1}{p^{l_{2}}+1}+2, \\ |D|&\le q-|A|+\max {\biggl \{1,\frac{|A|-1-(q-|A|)\max {\{0,|A|+p^{l_{1}}-q-1\}}}{p^{l_{1}}-1}\biggr \}}. \end{aligned}$$

A little notational comment: if $l_{1}=0$ we consider the upper bound trivial (but the lower bound becomes $({|A|+3})/{2}$, which is quite strong, identical to Szőnyi’s bound for $\mathbb {F}_{p}$).

Before we go to the proof, let us spend a few more words comparing this result with the one in [7]: bounds there are written as $({|A|-1})/({t+1})+2\le |D|\le ({|A|-1})/({s-1})$, for some appropriately defined *s*, *t*. The lower bound is the same as the one presented here, as *t* and $p^{l_{2}}$ are defined in the same way. The situation for the upper bound is more interesting: we have $s\le t=p^{l_{2}}\le p^{l_{1}}$, because the authors define *s* looking at the multiplicities in $H_{y}(x)$ alone (see the proof below for details) instead of the whole $x^{q}+g_{y}(x)$, which also gives a stronger geometric meaning to their *s* than to our $l_{1}$; however, our upper bound tends to be stronger when |*A*| is fairly close to *q* and there is a gap between *s* and $p^{l_{1}}$ (which can happen, as observed in [7]).

### Proof

First of all, we can suppose $\infty \in D$. If this were not true, we could take any $d\in D\setminus \{0\}$ (*D* is nonempty for $|A|>1$, and $D=\{0\}$ concludes the theorem) and consider $A'$ made of points $(a-db,b)$ for any $(a,b)\in A$, which implies also that $|A'|=|A|$: such a set would span directions given by

$$\begin{aligned} \frac{b'-b}{a'-db'-a+db}=\frac{1}{({a'-a})/({b'-b})-d}, \end{aligned}$$

from which it is clear that the new set of directions $D'$ is as large as *D*, since equalities are preserved, and that moreover $\infty \in D'$.

Define $n\ge 0$ so that $|A|=q-n$. First, define the *Rédei polynomial*

$$\begin{aligned} H_{y}(x)=\prod _{i=1}^{q-n}(x+ya_{i}-b_{i})\in \mathbb {F}_{q}[x,y], \end{aligned}$$

where the product is over all the $(a_{i},b_{i})\in A$: it is a polynomial of degree $q-n$ in two variables (some authors, like in [3], define it as a homogeneous polynomial in three variables, but by ensuring that $\infty \in D$ we do not need to do so). The usefulness of such polynomial lies in the fact that two points of *A* sitting on the same line with slope $y_{0}$ yield the same $x+y_{0}a-b$, so that a multiple root in $H_{y_{0}}(x)$ reflects the presence of a line with multiple points, i.e., a secant of *A*, and indicates that $y_{0}\in D$. We also define another function in two variables,

2.1 $$\begin{aligned} f_{y}(x)=\sum _{j=0}^{n}(-1)^{j}\sigma _{j}\bigl (\mathbb {F}_{q}\setminus \{ya_{i}-b_{i}|(a_{i},b_{i})\in A\}\bigr ) x^{n-j}, \end{aligned}$$

where $\sigma _{j}(S)$ is the *j*-th elementary symmetric polynomial of the elements in the set *S*; $f_{y}(x)$ is itself a polynomial in two variables (see [18, Thm. 4] for a recursive definition of $f_{y}(x)$), in which the coefficient of $x^{n-j}$ has *y*-degree *j*: therefore we can write

$$\begin{aligned} x^{q}+g_{y}(x)=H_{y}(x) f_{y}(x)\in \mathbb {F}_{q}[x,y], \end{aligned}$$

where $g_{y}(x)$ is a polynomial in two variables of *x*-degree $\le q-1$.

Substituting $y=y_{0}$ for some $y_{0}\notin D$, we observe that by definition the set $\mathbb {F}_{q}\setminus \{y_{0}a_{i}-b_{i}\mid (a_{i},b_{i})\in A\}$ has *n* elements and that $f_{y_{0}}(x)$ is simply the product of the $x-k_{i}$ for all the $k_{i}\in \mathbb {F}_{q}$ not counted in $H_{y_{0}}(x)$, so $g_{y_{0}}(x)=-x$: this means that the coefficients of $x^{q-1},x^{q-2},\ldots ,x^{|D|}$ in $g_{y}(x)$ are polynomials of degree $\le q-|D|$ in *y* that take value 0 for the $q-|D|+1$ values $y_{0}\in \mathbb {F}_{q}\setminus D$. Thus, these coefficients are the zero polynomial; in other words, the *x*-degree of $g_{y}(x)$ is at most $|D|-1$.

Working with *x*, *y* has allowed us to give a bound on the degree of $g_{y}(x)$. From now on, for the sake of simplicity we substitute one value $y\in D\setminus \{\infty \}$ inside our polynomials and drop the index, and we will work with only one variable; this is possible unless $D=\{\infty \}$, from which $|D|=1$ and *A* is contained in a vertical line.

Call $l_{2}$ the largest integer for which $g(x)\in \mathbb {F}_{q}[x^{p^{l_{2}}}]$: by the fact that any $x\mapsto x^{p^{i}}$ is an automorphism of $\mathbb {F}_{q}$, we have $g(x)=(\tilde{g}(x))^{p^{l_{2}}}$ for some $\tilde{g}(x)\in \mathbb {F}_{q}[x]\setminus \mathbb {F}_{q}[x^{p}]$. Decompose $x^{q}+g(x)$ into its irreducible factors, and call $l_{1}$ the largest integer for which $p^{l_{1}}$ divides the multiplicity of each linear factor (hence $l_{1}\ge l_{2}$): $l_{1},l_{2}$ depend on our choice of *y*, so for our definition we suppose that we have chosen a *y* that yields the smallest $l_{1}$. We can write

$$\begin{aligned} x^{q/p^{l_{2}}}+\tilde{g}(x)=(R(x))^{p^{l_{1}-l_{2}}}N(x), \end{aligned}$$

where $R(x)\in \mathbb {F}_{q}[x]\setminus \mathbb {F}_{q}[x^{p}]$ is such that $(R(x))^{p^{l_{1}}}$ is the divisor of $x^{q}+g(x)$ made of its linear factors (the fully reducible part of $x^{q}+g(x)$) and $N(x)\in \mathbb {F}_{q}[x]\setminus \mathbb {F}_{q}[x^{p}]$ is such that $(N(x))^{p^{l_{2}}}$ is the divisor of $x^{q}+g(x)$ made of its nonlinear factors. Note that $(N(x))^{p^{l_{2}}}$ must be a divisor of *f*(*x*). If $l_{1}=e$ then $x^{q}+g(x)=(x+c)^{q}$ for some $c\in \mathbb {F}_{q}$, which means that all the points of *A* lie on a line of slope equal to the *y* we have fixed, contradicting $\infty \in D$: hence $l_{2}\le l_{1}<e$.

Call $R^{*}(x)$ the divisor of *R*(*x*) made of all the irreducible factors of *R*(*x*) counted without multiplicity: $R^{*}(x)$ divides also $x^{q}-x$ by definition, so it divides $x^{q}+g(x)-(x^{q}-x)=g(x)+x\ne 0$ ($y\in D$ prevents us from having $g(x)=-x$). If an irreducible polynomial $k_{1}(x)$ divides another $k_{2}(x)$ with multiplicity *m* then it divides $k_{2}'(x)$ with multiplicity $m-1$, so

$$\begin{aligned} \frac{(R(x))^{p^{l_{1}-l_{2}}}}{R^{*}(x)}\;\bigg |\;(x^{q/p^{l_{2}}}+\tilde{g}(x))'=\tilde{g}'(x)\ne 0, \end{aligned}$$

where the last inequality is true because $\tilde{g}(x)\notin \mathbb {F}_{q}[x^{p}]$. From the reasoning above, we obtain

$$\begin{aligned} x^{q}+g(x)=\biggl (R^{*}(x)\cdot \frac{(R(x))^{p^{l_{1}-l_{2}}}}{R^{*}(x)}\biggr )^{\!p^{l_{2}}}\!\!\!(N(x))^{p^{l_{2}}}\;\bigg |\;(g(x)+x)^{p^{l_{2}}}(\tilde{g}'(x))^{p^{l_{2}}}f(x)\ne 0, \end{aligned}$$

and therefore

$$\begin{aligned} q=\deg {(x^{q}+g(x))}\le p^{l_{2}}(\deg {(g(x)+x)}+\deg \tilde{g}'(x))+\deg f(x); \end{aligned}$$

we have already determined that $\deg (g(x)+x)\le \deg g(x)\le |D|-1$, and similarly

$$\begin{aligned} \deg \tilde{g}'(x)\le \frac{\deg g(x)}{p^{l_{2}}}-1\le \frac{|D|-1}{p^{l_{2}}}-1, \end{aligned}$$

whence from the definition of *f*(*x*) we get

$$\begin{aligned} q \le p^{l_{2}}\biggl (|D|-1+\frac{|D|-1}{p^{l_{2}}}-1\biggr )+n\qquad \Longrightarrow \qquad |D|\ge \frac{q-n-1}{p^{l_{2}}+1}+2, \end{aligned}$$

settling the lower bound.

Let us focus now on the upper bound. Fix a point $(a,b)\in A$ and take a slope $y_{0}\in \mathbb {F}_{q}$: the multiplicity of the linear factor $x+y_{0}a-b$ inside *H*(*x*) determines how many points of *A* sit on the line defined by (*a*, *b*) and $y_{0}$. We know that the multiplicity of every linear factor in the whole *H*(*x*)*f*(*x*) is a multiple of $p^{l_{1}}$ and that it is at least 1 for this particular linear factor, since (*a*, *b*) sits on the line; however, we need a way to keep under control the number of false positives that come from the fully reducible part of *f*(*x*) (inexistent “ghost points” that make us overcount the contribution of a single line to *A*, and thus undercount |*D*|). The way to go is to bound the number of lines passing through (*a*, *b*) for which ghost points exist.

Let $f_{y}(x)$ be as in (2.1), call it for simplicity $f_{y}(x)=\sum _{j=0}^{n}\sigma _{y,j}x^{n-j}$ where the $\sigma _{y,j}$ are polynomials in *y* of degree *j*. Assume that $|D|<q+1$: then there will be a direction $y_{0}\in \mathbb {F}_{q}\setminus D$, as $\infty \in D$. For this $y_{0}$, $H_{y_{0}}(x) f_{y_{0}}(x)=x^{q}-x$ and $x+y_{0}a-b$ has multiplicity 1 in it; moreover, it must come from our fixed point (*a*, *b*), which means that it must divide $H_{y_{0}}(x)$ and be coprime with $f_{y_{0}}(x)$: this fact implies that the two-variable linear polynomial $x+ya-b$ cannot divide $f_{y}(x)$. In other words, we cannot write

2.2 $$\begin{aligned} (x^{n-1}+\tau _{y,1}x^{n-2}+\ldots +\tau _{y,n-1})(x+ya-b)=x^{n}+\sigma _{y,1}x^{n-1}+\ldots +\sigma _{y,n}\nonumber \\ \end{aligned}$$

for any choice of polynomials $\tau _{y,i}$; however, defining

$$\begin{aligned} \tau _{y,i}=\sum _{j=0}^{i}(-1)^{j}(ya-b)^{j}\sigma _{y,i-j} \end{aligned}$$

(here $\sigma _{y,0}=1$) we can ensure that the equality (2.2) works at least at the level of the coefficients of $x,x^{2},\ldots ,x^{n-1}$, which means that we must have

2.3 $$\begin{aligned} \sum _{j=0}^{n}(-1)^{j}(ya-b)^{j}\sigma _{y,n-j}\ne 0, \end{aligned}$$

so as to violate (2.2) for the free coefficient.

Every time $f_{y_{i}}(x)$ has a $x+y_{i}a-b$ factor (or, geometrically speaking, every time the line determined by (*a*, *b*) and $y_{i}$ has a ghost point), (2.2) is true for $y=y_{i}$ though, and in particular the LHS of (2.3) is indeed 0: that expression is a polynomial in *y* of degree *n*, so if there were $n+1$ lines with ghost points (2.3) would not be true, contradicting the fact that $x+ya-b$ cannot divide $f_{y}(x)$ as polynomials in two variables. Hence, at most *n* non-vertical lines through (*a*, *b*) have ghost points.

If $|D|-1\le n$ the upper bound stated in the theorem is already true, so suppose that the opposite holds: then there is a non-vertical line through (*a*, *b*) whose slope is in *D* with no ghosts. We can transform *A* as at the beginning of the proof to make that slope $\infty$, i.e., (*a*, *b*) lies on a vertical secant of *A*.

Each non-vertical line through (*a*, *b*) whose slope is in *D* has a multiple of $p^{l_{1}}$ among true points of *A* and ghost points ($l_{1}$ has been defined so as to make that statement true for all slopes at the same time). On the ghost-free lines there are at least $p^{l_{1}}-1$ true points besides (*a*, *b*), while on the ones with ghosts we can only say that there are at least $\max {\{0,p^{l_{1}}-1-n\}}$ of them (as the *x*-degree of $f_{y}(x)$ is *n*); finally, the vertical secant has at least $p^{l_{1}}$ points including (*a*, *b*), as we made sure that it had no ghosts before the transformation. Combining all of this with the bound on the number of lines with ghosts, and counting all the points of *A*, we get

$$\begin{aligned} (|D|-1-n)(p^{l_{1}}-1)+n\max {\{0,p^{l_{1}}-1-n\}}+p^{l_{1}}\le q-n. \end{aligned}$$

As we remarked after the statement of the theorem, for $l_{1}=0$ there is no upper bound. For $l_{1}>0$, the inequality above concludes the proof. $\square$

Now that we have the lower bound provided by Theorem 2.2, we can proceed with the proof of the first main theorem. We retain the same notation as in the previous proof.

### Proof of Theorem 1.3

Suppose that $|D|\notin \{1,q+1\}$ (otherwise the theorem is already proven); fix a slope $y_{0}\ne \infty$ and consider the polynomial $R^{*}(x)$ defined as in the proof of Theorem 2.2. Let $\varepsilon >0$ be small enough, and let $q'=p^{{e}/{2}}-\varepsilon$ for *e* even and $q'=p^{({e-1})/{2}}$ for *e* odd.

If the degree of $R^{*}(x)$ is $\le q'$, the set *A* must be contained in $\le q'$ lines with slope $y_{0}$, which means that one of them (call it *L*) will have to contain $\ge {|A|}/{q'}$ points of *A*; since *A* is not contained in one line there must be also a point of *A* outside *L*, and each secant laid between this point and a point of $A\cap L$ has a different slope, so that $|D|\ge {|A|}/{q'}$: for *e* even it means $|D|>{|A|}/\!{\sqrt{q}}$, while for *e* odd it means $|D|\ge {|A|}/{p^{({e-1})/{2}}}$.

If $R^{*}(x)$ has degree $>q'$, then by the fact that $(R^{*}(x))^{p^{l_{1}}}$ divides $x^{q}+g(x)$ we must have $p^{l_{2}}\le p^{l_{1}}<{q}/{q'}$: regardless of whether *e* is even or odd, $p^{l_{2}}\le p^{\lfloor {e}/{2}\rfloor }$ since $l_{2}$ is an integer. Using the lower bound in Theorem 2.2 (which holds for our *A*), we have

$$\begin{aligned} |D|\ge \frac{|A|-1}{p^{\lfloor {e}/{2}\rfloor }+1}+2=\frac{|A|}{p^{\lfloor {e}/{2}\rfloor }}+2-\frac{|A|}{p^{\lfloor {e}/{2}\rfloor }(p^{\lfloor {e}/{2}\rfloor }+1)}-\frac{1}{p^{\lfloor {e}/{2}\rfloor }+1}. \end{aligned}$$

For *e* even, the bound above implies $|D|>{|A|}/\!{\sqrt{q}}$, while for *e* odd we can obtain $|D|\ge ({|A|+3})/({p^{({e-1})/{2}}+1})$. $\square$

## Growth in $\mathrm {Aff}(\mathbb {F}_{q})$

We move now to the proof of Theorem 1.4. We follow closely the proof of the analogous result in [16] for $\mathbb {F}_{p}$: the only difference is that we use Theorem 1.3 instead of Szőnyi’s bound, and that, as we have already said, we absorb the case of *A* of medium size into alternative (b), without resorting to Alon’s bound to fall into (c).

We remind the reader of two well-known and by now classical results. First, an inequality, deducible in multiple ways from bounds by Ruzsa (see for instance [17]), states that for any group *G* and any $A=A^{-1}\subseteq G$ the equality $|A^{3}|=C|A|$ implies $|A^{k}|\le C^{k-2}|A|$ for any $k\ge 4$. Second, Kneser’s theorem [11] tells us that, given any abelian *G* and any $A,B\subseteq G$, there is a proper subgroup *H* with $|A+B|\ge \min {\{|G|,|A|+|B|-|H|\}}$.

Theorem 1.3 and Lemma 2.1 will take care of small and medium |*A*|, respectively. For |*A*| large we will instead make use of the following bound, due to Vinh [21, Thm. 3]: the statement therein says actually something weaker, but it is based on a well-known graph-theoretic result [2, Cor. 9.2.5] which allows to be reformulated as follows (as [16] does for $\mathbb {F}_{p}$).

### Proposition 3.1

Let *q* be a prime power, let *P* be a set of points in $\mathbb {F}_{q}^{2}$, and let *L* be a set of lines in $\mathbb {F}_{q}^{2}$. Define *I*(*P*, *L*) as the set of pairs $(p,l)\in P\times L$ such that $p\in l$. Then

$$\begin{aligned} \biggl |I(P,L)-\frac{|P|\cdot |L|}{q}\biggr |\le \sqrt{q\cdot |P|\cdot |L|}\,. \end{aligned}$$

We note that the phenomenon described in Proposition 3.1 is in no way restricted to $\mathbb {F}_{q}^{2}$. The same graph-theoretic tools can be applied to incidences between points and linear hyperspaces in $\mathbb {F}_{q}^{n}$ [13, Cor. 2], and even more generally to incidences between points and blocks in BIBDs [13, Thm. 1]: Proposition 3.1 is a particular case of either of those.

Let us also give here separately a lemma that will provide the upper bounds on $\pi (A)$ in (b)–(c) of Theorem 1.4.

### Lemma 3.2

For any $$g=\begin{pmatrix}a&{}b\\ 0&{}1\end{pmatrix}\in \mathrm {Aff}(\mathbb {F}_q)\setminus \{\mathrm {Id}\}$$, define the map

$$\begin{aligned} \varphi _{g}:\mathrm {Aff}(\mathbb {F}_q)\rightarrow \mathrm {Aff}(\mathbb {F}_q), \qquad \varphi _{g}(h)=hgh^{-1}. \end{aligned}$$

Then

- any point in the image of $\varphi _{g}$ has as preimage a line of slope ${b}/({a-1})$;
- if $A=A^{-1}\subseteq \mathrm {Aff}(\mathbb {F}_{q})$ and $g\in A^{k}$ then $|\pi (A)|\le {|A^{k+3}|}/{|\varphi _{g}(A)|}$.

### Proof

(a) This is just an easy computation: as

$$\begin{aligned} \begin{pmatrix}r&{}s\\ 0&{}1\end{pmatrix}\begin{pmatrix}a&{}b\\ 0&{}1\end{pmatrix} \begin{pmatrix}r^{-1}&{}-r^{-1}s\\ 0&{}1\end{pmatrix}= \begin{pmatrix} a &{} \quad br &{}{+}\, (1-a)s\\[2mm] 0 &{} &{} 1\end{pmatrix}, \end{aligned}$$

two elements are in the preimage of a single point if and only if $br+(1-a) s=br'+(1-a) s'$, from which all pairs of elements with $({s'-s})/({r'-r})={b}/({a-1})$ must be sent to the same point by $\varphi _{g}$ (we allow $a=1$ and a slope equal to $\infty$, but we avoid $(a,b)=(1,0)$ since $g\ne \mathrm {Id}$).

(b) On one hand we have $|A\varphi _{g}(A)g^{-1}|=|A\varphi _{g}(A)|\le |A^{k+3}|$, while on the other hand any element of $A\varphi _{g}(A)g^{-1}$ is of the form $a_{1}a_{2}ga_{2}^{-1}g^{-1}\in AU$: since

$$\begin{aligned} \begin{pmatrix}x&{}y\\ 0&{}1\end{pmatrix} \begin{pmatrix}1&{}z\\ 0&{}1 \end{pmatrix}=\begin{pmatrix} x &{}\quad xz &{}+y\\ 0 &{}&{} 1\end{pmatrix}, \end{aligned}$$

pairs in $A\times U$ with either distinct *x* or with the same *x*, *y* but distinct *z* will all give different products in *AU*; hence we can select one element of *A* for each value of *x* (therefore $|\pi (A)|$ of them) and all the $a_{2}ga_{2}^{-1}g^{-1}$ ($|\varphi _{g}(A)|$ of them, they are all multiplied by the same $g^{-1}$), and obtain the other side of the bound, namely $|A\varphi _{g}(A) g^{-1}|\ge |\pi (A)|\cdot |\varphi _{g}(A)|$. $\square$

With these tools at our disposal, we can proceed with the proof.

### Proof of Theorem 1.4

Let us start with the case of *A* large: fix a constant $c>1$ to be chosen later and impose $|A|=c'q$ with $c'\ge c$. We use the bound from Proposition 3.1 with $P=A$ and $L=\overline{L(A)}$ (the set of lines that are not determined by *A*), interpreted as a lower bound on the expression inside the absolute value, and combine it with the trivial observation that $I(A,\overline{L(A)})\le |\overline{L(A)}|$ by the definition of $\overline{L(A)}$: this yields

$$\begin{aligned} |\overline{L(A)}|\le q^{2}\frac{c'}{(c'-1)^{2}} \le q^{2}\frac{c}{(c-1)^{2}}\quad \ \Longrightarrow \quad \ |L(A)|\ge q+q^{2}\biggl (1-\frac{c}{(c-1)^{2}}\biggr ). \end{aligned}$$

If we choose here

$$\begin{aligned} c=1+\frac{1+\sqrt{3-{2}/{p}}}{1-{1}/{p}} \end{aligned}$$

(or $c=3+2\sqrt{2}$, which is a choice valid for all primes *p*), by the pigeonhole principle there must exist $\ge {q} (1+{1}/{p})/2$ non-vertical lines of *L*(*A*) parallel to each other; call *d* the direction defined by such lines. Given any two elements of *A* sitting on one of these lines, we have

$$\begin{aligned} g=\begin{pmatrix}a_{1}&{}b_{1}\\ 0&{}1\end{pmatrix}^{-1}\begin{pmatrix}a_{2}&{}b_{2}\\ 0&{}1\end{pmatrix}= \begin{pmatrix} a_{2}a_{1}^{-1} \quad \quad &{}b_{2}a_{1}^{-1} &{}-b_{1}a_{1}^{-1} \\ 0&{}&{} 1\end{pmatrix}=\begin{pmatrix}a'&{}b'\\ 0&{}1\end{pmatrix}, \end{aligned}$$

with ${b'}/({a'-1})=({b_{2}-b_{1}})/({a_{2}-a_{1}})=d$, so by Lemma 3.2 (a) there are at least ${q} (1+{1}/{p})/2>{q}/{2}$ elements in $\varphi _{g}(A)$; by Lemma 3.2 (b) and Ruzsa’s inequality, this implies that $|\pi (A)|<{2|A^{5}|}/{q}\le {2}C^{3}|A|/q$. Moreover, the unipotent subgroup *U* is isomorphic to $\mathbb {F}_{q}$ as an additive group, so that its largest proper subgroup is of size *q*/*p*; therefore, since $\varphi _{g}(A) g^{-1}\subseteq A^{6}\cap U$ has $|\varphi _{g}(A) g^{-1}|\ge {q} (1+{1}/{p})/2$, by Kneser’s theorem we must have

$$\begin{aligned} A^{8}\supseteq AgAAg^{-1}A\supseteq (\varphi _{g}(A) g^{-1})(\varphi _{g}(A) g^{-1})^{-1}\supseteq U, \end{aligned}$$

and we fall into case (c) of the theorem.

Let us deal now with *A* of medium size: suppose $q\!<\!|A|\!<\!cq$, so that by Lemma 2.1 every direction is determined by some pair of points of *A*. Partition $A^{2}\setminus \{\mathrm {Id}\}$ into $q+1$ subsets, collecting into each one of them elements having the same value for ${b}/({a-1})\in \mathbb {F}_{q}\cup \{\infty \}$. Every two distinct $a_{1},a_{2}\in A$ yield an element $a_{1}^{-1}a_{2}\in A^{2}$ that is located inside the part corresponding to the slope of the line they define: by the pigeonhole principle there will be a part (identifiable with some $d\in \mathbb {F}_{q}\cup \{\infty \}$) with at most $({|A^{2}|-1})/({q+1})$ elements, and therefore every line of *L*(*A*) in the direction *d* must have at most $({|A^{2}|-1})/({q+1})+1$ elements of *A* on it, since $a_{1}^{-1}a_{i}\ne a_{1}^{-1}a_{j}$ for $a_{i}\ne a_{j}$. We have thus given a bound on the number of points of *A* sent to the same element by the map $\varphi _{g}$ for some $g\in A^{2}$ with ${b}/({a-1})=d$, which translates to

$$\begin{aligned} |\varphi _{g}(A)|\ge \frac{(q+1)|A|}{|A^{2}|+q}>\frac{|A|}{Cc+1}; \end{aligned}$$

proceeding as before, by Lemma 3.2 (b) and Ruzsa’s inequality we conclude that $|\pi (A)|<(Cc+1) C^{3}\le (4+2\sqrt{2}) C^{4}$ and we reach case (b) of the theorem.

For *A* small (i.e., $1<|A|\le q$) we repeat essentially what we did for *A* medium, but instead of $|D|=q+1$ we use the bounds in Theorem 1.3 on the number of directions |*D*| spanned by *A*. We obtain

$$\begin{aligned} |\varphi _{g}(A)|>\frac{|A|^{2}}{q'(|A^{2}|-1)+|A|}>\frac{|A|}{Cq'+1}, \end{aligned}$$

where $q'=\sqrt{q}$ for *e* even and $q'=p^{({e-1})/{2}}+1$ for *e* odd, from which we get $|\pi (A)|<(p^{\lfloor {e}/{2}\rfloor }+2) C^{4}$ and end up in case (b). Finally, we need to deal with the other alternative in Theorem 1.3, namely that *A* may be contained in one line: in other words, the elements of *A* are either all of the form $(a,ad+b)$ for some $b,d\in \mathbb {F}_{q}$, through the identification of $\mathrm {Aff}(\mathbb {F}_{q})$ with $\mathbb {F}_{q}^{*}\times \mathbb {F}_{q}$, or all contained in *U*. In the latter alternative $A\subseteq U$ implies $|\pi (A)|=1$, yielding (b); in the former, since $A=A^{-1}$ and $(a,ad+b)^{-1}=(a^{-1},-a^{-1}b-d)$, we must have $b=-d$ and then $A\subseteq \mathrm {Stab}(-d)$. $\square$

## Concluding Remarks

Theorem 1.4, as addressed several times, gives a structural result on $\mathrm {Aff}(\mathbb {F}_{q})$; more than that, it shows that the techniques used for the case of $\mathbb {F}_{p}$ in [16] can be generalized to arbitrary finite fields. This is not a new concept, as the sum-product theorem has also followed an analogous trajectory, although the same cannot be said of the proofs about the affine group specifically. There is value in such feats, as it has often been the case that results for $\mathbb {F}_{q}$ have followed the $\mathbb {F}_{p}$ case once the machinery had been understood and streamlined; in some situations it is yet an ongoing process, for example with [8] still missing an equally strong counterpart in $\mathbb {F}_{q}$ (where weakened generalizations are included however in [4, 15]).

It is the hope of the author that the present result provides another small tessera in the mosaic of growth in matrix groups. The differences between Theorems 1.2 and 1.4 seem also to reflect the general divergence point between prime fields and general finite fields: the obstacle presented by $\sqrt{q}$ is in substance the same as providing that we avoid proper subfields, for its origin lies in the complications of the Frobenius map $x\mapsto x^{p}$ in the course of the proof of Theorem 2.2. In this sense, the result also works as a reaffirmation of deeply rooted principles that are likely to resurface in future research on similar cases, involving matrix groups having larger rank and a more complicated structure.

In particular, this refers to the example (immediately following Theorem 1.4) of a set *A* whose growth is stifled by a subfield. The question of whether the example we provided is essentially the only one, in line with the structure predictions of the Helfgott–Lindenstrauss conjecture [9, Conj. 1], is not answered here. However, the methods involved in the proof of Theorem 1.4 seem to yield themselves to be employed in such a task: having a power of *p* as a factor in the first half of Theorem 1.4(b) translates into a condition on the polynomials describing the points of *A* as being polynomials in some power $x^{p^{l}}$, instead of simply polynomials in *x*. This in turn might provide enough information on the arrangement of the points of *A* in the affine plane (arrangement that defines $H_{y}(x)$, essentially) to say that *A* must necessarily be “stuck in a subfield”. This avenue of inquiry would show a quantitative version of the aforementioned conjecture for the case of $\mathrm {Aff}(\mathbb {F}_{q})$, and it is worth exploring.
